# Radiographic assessment of endodontic mishaps in an undergraduate student clinic: a 2-year retrospective study

**DOI:** 10.7717/peerj.13858

**Published:** 2022-08-04

**Authors:** Manal Matoug-Elwerfelli, Ahmed Abdou, Wejdan Almutairi, Malak Alhuthayli, Shaikhah Aloyaynaa, Rahaf Almohareb

**Affiliations:** 1 College of Dental Medicine, QU Health, Qatar University, Doha, Qatar; 2 Prosthetic Dentistry Department, Division of Biomaterials, Faculty of Dentistry, King Salman International University, El Tur, South Sinai, Egypt; 3 College of Dentistry, Princess Nourah Bint Abdulrahman University, Riyadh, Saudi Arabia; 4 Department of Clinical Dental Sciences, College of Dentistry, Princess Nourah bint Abdulrahman University, Riyadh, Saudi Arabia

**Keywords:** Dental students, Dentistry, Education, Endodontics, Instrumentation

## Abstract

**Objectives:**

The aim of this study was to compare the occurrence of instrumentation and obturation related endodontic procedural mishaps following the use of either, stainless steel hand or engine-driven rotary instrumentation techniques.

**Methods:**

From a computerized hospital database, a total of 730 dental patient records who had received endodontic treatment by undergraduate dental students between August 2018 to September 2020 were retrieved. The inclusion criteria were primary (non-surgical) endodontic treatment on permanent teeth with complete radiographic records. Following record screening, a final sample of *n* = 475 dental records were included. Radiographic records were evaluated for both instrumentation and obturation related mishaps. The data was analysed using multiple logistic regression analysis (*α* = 0.05).

**Results:**

Engine-driven rotary instrumentation resulted in a significant decrease in the overall occurrence of instrumentation related endodontic mishaps by 40% compared to hand instrumentation (Odds Ratio = 0.59 [0.36–0.97], *p* = 0.04). In particular, rotary instrumentation decreased ledge formation, perforation and obturation related mishaps, with minimal effect on the limitation of zipping.

**Conclusion:**

The use of rotary instrumentation techniques may reduce the incidence of instrumentation and obturation endodontic mishaps in the undergraduate dental clinic.

## Introduction

Over the past decades, dentistry has undergone several advancements in terms of instruments and materials. Specifically, the field of endodontics has shown major innovation from the traditional use of hand stainless steel (SS) files to the nickel-titanium (NiTi) engine-driven instrumentation techniques, utilizing either rotational or reciprocating kinetics (Peters, 2004; Çapar & Arslan, 2016). NiTi alloy files gained significant attention due to inherent material advantages such as corrosion resistance, shape-memory, and super-elastic properties (Gavini et al., 2018; Tabassum, Zafar & Umer, 2019).

The utilization of SS hand files has been the traditional gold standard files for both pre-clinical and clinical training both locally and internationally. Unfortunately, a higher frequency of endodontic mishaps such as instrument separation, ledging, and root canal perforation following the use of SS hand files within undergraduate teaching clinics are reported (Balto et al., 2010; AlRahabi, 2017; Hamid et al., 2018). These clinical unwanted mishaps are most likely related to multiple variables including the stiff (non-flexible) nature of the SS alloy properties. Therefore, to overcome these mishaps, the shift towards the enhanced elasticity and flexibility of NiTi alloys was welcomed.

Following their acceptance, NiTi engine-driven files have been gradually introduced to the undergraduate dental curriculum; within the pre-clinical setting and the undergraduate clinical training (Martins et al., 2012; Al Raisi, Dummer & Vianna, 2019). Enhanced undergraduate dental students perception, improved self-confidence and clinical experience following the use of NiTi engine-driven instrumentation, in comparison to SS hand instrumentation was reported (Martins et al., 2012).

Within the hands of the inexperienced operator (undergraduate dental students), the majority of studies conducted either on NiTi files alone or in comparison to SS files are performed *in vitro* (Alves et al., 2013; Kwak et al., 2016; Alemam, Dummer & Farnell, 2017), with variable *in vivo* studies of significant methodology variation (Cheung & Liu, 2009; Abu-Tahun et al., 2014; Bruno et al., 2016). Therefore, the aim of this study was to compare the occurrence of endodontic procedural mishaps following the use of two instrumentation techniques: SS hand K-files and NiTi rotary files in an undergraduate student clinic. Furthermore, to determine the correlation of endodontic mishaps with multiple variables; tooth position (anterior or posterior), instrumentation technique (hand or rotary), operator (4^th^ or 5^th^ year undergraduate students), number of canal(s) (1, 2, 3, or 4 canals), and the degree of root curvature (mild, moderate, or severe). The null hypotheses were that: (1) no significant difference in the occurrence of endodontic instrumentation and obturation related mishaps between SS hand files and NiTi rotary instrumentation, and (2) no correlation between the endodontic mishaps and the tested variables.

## Materials & Methods

Ethical approval for this study was approved by Princess Nourah Bint Abdulrahman University (PNU) in Riyadh, Saudi Arabia, Institutional Review Board Committee (approval no. 20-0183).

Due to the retrospective nature of this study, patient consent was waived as data were already available as part of routine hospital procedures. Furthermore, the investigators ensured all data was anonymized without any reference to patients’ identity.

A total of 730 dental patient records who had received endodontic treatment at the College of Dentistry Teaching Clinics, PNU between August 2018 to September 2020 were retrieved from a computerized hospital database AxiUm (Exan Group, Coquitlam, Canada). The inclusion criteria were completed primary (non-surgical) endodontic treatment on permanent teeth with complete radiographic records (a minimum of three good quality periapical radiographs: a preoperative, working length and postoperative radiographs). Permanent teeth with uncompleted endodontic treatment, retreatment cases, and endodontic treatment on deciduous dentition, immature permanent teeth or performed by endodontic residents were excluded. Dental records with missing or poor quality (undiagnostic) radiographs were also excluded.

All endodontic treatments were carried out by 4^th^ and 5^th^ year undergraduate students using either SS hand or engine-driven ProTaper Universal (PTU) files (Dentsply Maillefer, Ballaigues, Switzerland) followed by lateral condensation obturation. Endodontic clinical staff supervised all treated cases, with an average staff to student ratio of 1:7. An aseptic technique with rubber dam isolation was mandatory in all clinical cases. Root canal treatment followed standard clinical steps, starting with access cavity and orifice enlargement, utilizing either Gates-Glidden drills (Dentsply Maillefer, Ballaigues, Switzerland) or orifice openers SX-PTU prior to hand and rotary instrumentation, respectively. Root canal instrumentation was performed with the step-back technique using with either SS hand K-files 0.02 taper (Mani, Tochigi, Japan) for hand instrumentation technique or PTU files for engine-driven instrumentation technique, as per manufacture sequence. Additionally, irrespective to the instrumentation technique, a standard glide-path to the full working length was established with SS K-file, mainly of size 10 or 15, with the aid of 17% Ethylenediaminetetraacetic acid (EDTA) lubricant gel (MD-ChelCream Meta Biomed, Korea), as required. Working lengths were determined using apex locator Root ZX II (J. Morita, Tokyo, Japan) and intra-oral digital periapical radiographs. The size of the initial file was determined as the first SS K-file to engage the canal passively to its entire working length, in the majority of the included cases K-file size 15 was the most appropriate. In both instrumentation groups’, standard clinical protocols such as recapitulation (patency) file between each successive file, copious canal irrigation with 2.25% sodium hypochlorite, 17% EDTA solution (Vista Dental, Racine, WA, USA), and saline as a final irrigation was performed. The master apical file size was usually determined as a minimum of three successive files larger than the initial file size. Canal obturation was carried out using lateral condensation technique with gutta percha cones and AH plus sealer (Dentsply Maillefer, Ballaigues, Switzerland).

Two calibrated examiners (experienced endodontists) were involved in data collection. Prior to the actual study, the examiners were calibrated by assessing a few selected cases with endodontic specialists. Inter- and intra- examiner reliability was determined by scoring 20 random radiographs. Radiographs were evaluated twice by the same examiners, the first stage involved inter-examiner reliability, followed by the intra-examiner reliability 4 weeks later. These radiographs were included in the main study.

Both examiners evaluated the presence of endodontic instrumentation mishaps and quality of canal obturation by noting the entries in the patients’ electronic records and careful interpretation of the radiographs. Mesial and distal radiographic angulations were also assessed for multi-rooted teeth. The tooth was considered as one unit, scored according to the presence of mishaps in any canal. The criteria for evaluation of instrumentation and obturation related endodontic mishaps were as previously described by Barrieshi-Nusair, Al-Omari & Al-Hiyasat (2004) and Eleftheriadis & Lambrianidis (2005), and summarized in Table 1. Demographic data and clinical related parameters such as; tooth position, instrumentation technique, operator, number of canal(s), and degree of root curvature were also extracted. The degree of root curvature was determined through MiPACS Dental Enterprise Viewer software and classified into three categories based on a modified Schneider classification (Schneider, 1971; Qiao et al., 2021). Category I; mild curvature of the root canal (curvature < 5°), category II; moderate curvature of the root canal (curvature 5 to <20°), and category III; severe curvature of the root canal (curvature ≥ 20°).

**Table 1:** Identification criteria for the evaluation of instrumentation and obturation related procedural mishaps.

Root canal treatment mishaps	Identification criteria
Instrumentation related mishaps	
Ledges	Identified when a file or obturation material did not follow the original anatomical curvature of the root canal
Zipping	Identified when the apical termination of the filled canal appeared as an elliptical shape transported to the outer wall
Instrument separation	Identified when a radiopaque separated instrument was detected in the radiograph
Perforation	Furcation perforation: identified when the extrusion of a file or obturation material through the furcation area in multi-rooted teeth
	Strip perforation: identified when the extrusion of a file or obturation material was detected on the lateral (inner) wall of the root in multi-rooted teeth
	Root perforation: identified when the extrusion a file or obturation material was detected in any area of the root including apical area (apical perforation), and excluding furcation and strip perforation
Obturation related mishaps	
Length of root canal filling	Acceptable: root filling ending within 2 mm short of the radiographic apex
	Unacceptable: under filling (root filling ending <2 mm short of radiographic apex) or over filling (root filling ending beyond the radiographic apex)
Density of root canal filling	Acceptable: uniform density of root filling without voids and canal space is not visible
	Unacceptable: poor density of root filling with the presence of voids and visible canal space

Statistical analysis of the data was performed using Stata (Version 16; StataCorp LLC, College Station, TX, USA). Inter- and intra-examiner reliability of the radiographic endodontic mishaps were measured using Cohen’s Kappa. The presence of endodontic mishaps in relation to tested variables; tooth position (anterior or posterior), instrumentation technique (hand or rotary), operator (4^th^ or 5^th^ year undergraduate students), number of canal(s) (one, two, three or or four canals), and root curvature (mild, moderate, or severe) were assessed. Multiple logistic regression analysis was performed to determine statistical significance and the association of operation variables on the presence of mishaps. A penalized likelihood-based method (Firth logistic regression) was performed as the presence of mishaps revealed data separation. The level of statistical significance of 0.05 was set for all analyses.

## Results

**Figure 1:**
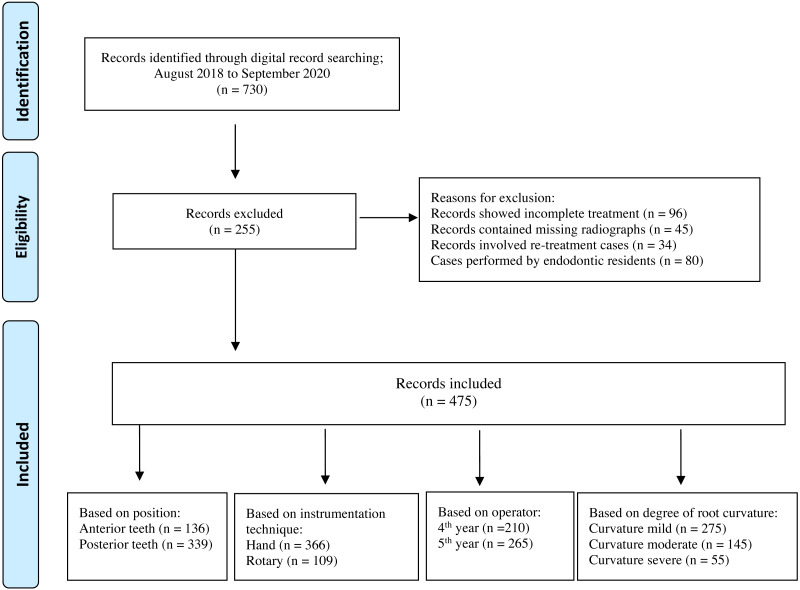
Flow diagram of included studies.

Results of the inter- and intra- examiner reliability of the radiographic scores showed a strong reliability, both, κ = 0.875 (95% confidence interval (CI) [0.638–1.000]). A total of *n* = 730 dental records were initially examined, all of which had received endodontic treatment at the College of Dentistry teaching clinics, PNU. Following record screening based on eligibility criteria, a final total of *n* = 475 root canal treated teeth were analysed (Fig. 1). The overall incidence of mishaps was *n* = 166 (45.4%) following hand instrumentation and *n* = 42 (38.5%) following engine-driven instrumentation. The overall distribution of mishaps in relation to tested variables is presented in Table 2.

**Table 2:** Incidence of mishaps (n[%]) according to tested variables; tooth position, instrumentation technique, operator, number of canals, and root curvature.

	**Tooth position**		**Instrumentation technique**		**Operator**		**Number of canal(s)**				**Root curvature**		
	**Anterior**	**Posterior**	**Hand**	**Rotary**	**4**th **year**	**5**th **year**	**1 canal**	**2 canals**	**3 canals**	**4 canals**	**Mild**	**Moderate**	**Severe**
**Ledge**	4[2.9]	16[4.7]	17[4.6]	3[2.8]	9[4.3]	11[4.2]	9[3.4]	4[4.6]	6[6.3]	1[3.8]	7[2.5]	10[6.9]	3[5.5]
**Zipping**	4[2.9]	13[3.8]	13[3.6]	4[3.7]	8[3.8]	9[3.4]	8[3]	3[3.4]	6[6.3]	0[0]	9[3.3]	7[4.8]	1[1.8]
**Perforation**	44[32.4]	125[36.9]	135[36.9]	34[31.2]	76[36.2]	93[35.1]	86[32.3]	29[33.3]	43[44.8]	11[42.3]	94[34.2]	57[39.3]	18[32.7]
**Instrument separation**	0[0]	13[3.8]	10[2.7]	3[2.8]	2[1]	11[4.2]	0[0]	5[5.7]	5[5.2]	3[11.5]	2[0.7]	3[2.1]	8[14.5]
**Overall instrumentation mishaps**	52[38.2]	156[46]	166[45.4]	42[38.5]	92[43.8]	116[43.8]	102[38.3]	39[44.8]	55[57.3]	12[46.2]	111[40.4]	70[48.3]	27[49.1]
**Overall obturation mishaps**	58[42.6]	160[47.2]	177[48.4]	41[37.6]	100[47.6]	118[44.5]	118[44.4]	46[52.9]	40[41.7]	14[53.8]	124[45.1]	65[44.8]	29[52.7]

Multiple logistic regression analysis showed that, both number of canals and instrumentation technique of the tested variables had a significant effect on the occurrence of instrumentation and obturation related endodontic mishaps. The use of engine-driven rotary instrumentation resulted in statistical significant decrease in the overall occurrence of instrumentation related mishaps by 40% compared to hand instrumentation with an Odds Ratio (OR) of 0.59 (*p* = 0.04). On the other hand, the number of canals (3 canals) increased the overall mishaps (OR = 2.3, *p* = 0.01) (Table 3).

**Table 3:** Logistic regression analysis showing the effect of tested variables on the occurrence of procedural mishaps.

	**Ledge**		**Zipping**		**Perforation**		**Overall instrumentation mishaps**		**Overall obturation mishaps**	
	**OR [95% CI]**	***p***-**value**	**OR [95% CI]**	***p***-**value**	**OR [95% CI]**	***p***-**value**	**OR [95% CI]**	***p***-**value**	**OR [95% CI]**	***p***-**value**
**Tooth position [Anterior]** ^a^										
**Posterior**	1.42[0.38-5.32]	0.6	1.22[0.31-4.85]	0.78	1.15[0.67-1.96]	0.61	1.17[0.69-1.97]	0.55	1.33[0.8-2.21]	0.28
**Root curvature [Mild]** ^a^										
**Moderate**	2.79[0.98-7.99]	0.06	1.11[0.36-3.45]	0.86	1.03[0.64-1.63]	0.91	1.12[0.72-1.76]	0.62	1.06[0.68-1.66]	0.79
**Severe**	2.48[0.57-10.84]	0.23	0.57[0.08-3.94]	0.57	0.66[0.33-1.34]	0.25	1.04[0.54-2.03]	0.90	1.51[0.78-2.93]	0.22
**Number of canals [Single canal]** ^a^										
**2 canals**	0.98[0.27-3.56]	0.97	1.19[0.29-4.97]	0.81	1.01[0.57-1.79]	0.98	1.19[0.68-2.07]	0.52	1.21[0.7-2.09]	0.49
**3 canals**	1.17[0.32-4.25]	0.81	2.37[0.59-9.58]	0.23	2.05[1.12-3.78]	0.02^*^	2.3[1.27-4.19]	0.01^*^	0.83[0.46-1.5]	0.53
**4 canals**	1.02[0.14-7.29]	0.99	0.74[0.04-15.35]	0.85	2.01[0.81-4.98]	0.13	1.49[0.61-3.64]	0.38	1.26[0.52-3.06]	0.61
**Operators** [**4thyear**]^a^										
**5**th **year**	0.91[0.33-2.49]	0.85	0.76[0.25-2.29]	0.63	0.89[0.57-1.39]	0.6	0.88[0.57-1.36]	0.57	0.86[0.56-1.32]	0.49
**Instrumentation technique [Hand]** ^a^										
**Rotary**	0.47[0.13-1.62]	0.23	0.98[0.29-3.34]	0.97	0.65[0.39-1.08]	0.1	0.59[0.36-0.97]	0.04^*^	0.64[0.39-1.04]	0.07

**Notes.**

CI confidence interval OR Odds Ratio (OR <1; decreases the occurrence of mishaps, OR = 1; no effect on mishaps, OR >1; increases the occurrence of mishaps)

a Reference variable for each parameter.

* Statistically significant (*p* < 0.05).

In regard to instrumentation related mishaps, multiple logistic regression analysis showed that none of the tested variables had a significant effect on the occurrence of ledges and zipping. Ledges were seen in 17 (4.6%) and three (2.8%) cases following hand and engine-driven instrumentation, respectively. Of these, four (2.9%) cases were related to anterior teeth and 16 (4.7%) in posterior teeth (Table 2). However, increasing root curvature < 20 might be correlated with a higher incidence on the ledge formation (OR = 2.79, *p* = 0.06) (Table 3).

The occurrence of zipping followed a similar pattern to ledging and were seen in 13 (3.6%) and four (3.7%) cases following hand and engine-driven instrumentation, respectively. Of these, four (2.9%) cases were related to anterior teeth and 13 (3.8%) in posterior teeth (Table 2).

Furcation, strip, and root perforations were seen in one, four, and 164 cases, respectively. Out of the 164 cases of root perforation, the majority were apical root perforation seen in cases of over-instrumentation. Due to the small number of furcation and strip perforations, all types of perforation were grouped together as “perforation mishaps”. A total of 135 (36.9%) perforations were associated with hand instrumentation in contrast to 34 (31.2%) related to engine-driven instrumentation. Of these 44 (32.4%) were seen in anterior teeth and 125 (36.9%) in posterior teeth (Table 2). Multiple logistic regression analysis showed that occurrence of perforation was significantly higher in three canals compared to single canal teeth with a 200% incidence of increasing the risk of perforation (OR = 2.05, *p* = 0.02) (Table 3). The detailed distribution of different type of perforations for each tooth are presented in the Table S1.

Instrument separation was found in *n* = 13 cases, with an incidence of 10 (2.7%) and 3 (2.8%) related to hand and engine-driven instrumentation, respectively. Furthermore, an incidence of instrument separation resulted for higher degree of canal curvature, with an incidence of 2 (0.7%), 3 (2.1%), and 8 (14.5%) related to a curvature of mild, moderate, and severe, respectively.

Both length and density of root canal filling were grouped together as obturation related mishaps. Multiple logistic regression analysis showed that none of the tested variables had a significant effect on the occurrence of obturation mishaps. A higher occurrence of unacceptable rated obturation quality was seen following hand (48.4%) in comparison to engine-driven (37.6%) instrumentation techniques (Table 2). In terms of the operator level, no statistical significance difference was seen in regards to undergraduate student level (4^th^ or 5^th^ year) and the association of endodontic mishaps (Table 3).

Overall, compared to hand instrumentation, engine-driven rotary instrumentation decreased overall instrumentation and obturation related mishaps by 41% (OR = 0.59, *p* = 0.04) and 36% (OR = 0.64, *p* = 0.07), respectively. Specifically, decreasing the ledge formation by 50% (OR = 0.47, *p* = 0.23), perforation by 35% (OR = 0.65, *p* = 0.1). However, the effect on zipping was limited (OR = 0.98, *p* = 0.97) (Table 3). The detailed distribution of ledge formation, zipping, perforation, and obturation related mishaps for each tooth are presented in the Table S2.

The power of the study has been calculated after completion of the study and found to be 99.9% with the examined sample size of 475 to detect an Odds Ratio (O1/O0) of 0.59 for the overall instrumentation and obturation related mishaps with a significance level of 0.05. A two-sided exact test was used and assumed that the population proportion under the null hypotheses (P0) is 0.5.

## Discussion

Undergraduate endodontic education within dental schools has evolved, and indeed improved, throughout the years. This has been largely driven by enhanced educational teaching methods and advancements in endodontic materials and equipment (Al Raisi, Dummer & Vianna, 2019). Engine-driven endodontic motors have become a popular endodontic armamentarium, enabling faster and easier instrumentation of the root canal (Schäfer, Schulz-Bongert & Tulus, 2004). Furthermore, the unique properties of NiTi alloy files such as biocompatibility, greater strength, low modulus of elasticity, enhanced flexibility, and super-elasticity are advantageous in comparison to their predecessors, SS alloy (Gavini et al., 2018; Tabassum, Zafar & Umer, 2019).

Currently, numerous NiTi rotary systems are available on the dental market. Of these, 2^nd^ generation ProTaper rotary instruments are one of the most extensively studied systems (Hieawy et al., 2015; Martins et al., 2020). The PTU rotary system was used in this study as they are the main rotary system available for undergraduate students at PNU dental clinic, for standardization of treatment quality. PTU files are manufactured from conventional NiTi alloy with a progressive taper design, a non-radial land design, convex triangular cross section, and a non-cutting safety tip (Hieawy et al., 2015; Martins et al., 2020). These design modifications are sought to enhance file flexibility, reduce cyclic fatigue, and improve torsional resistance failure, thus decreases instrument fracture rate (Bruno et al., 2016; Alqedairi et al., 2019). Therefore, ensuring safety clinical usage particularly with undergraduate students (Bruno et al., 2016). Contemporary endodontic teaching methods, during pre-clinical and clinical training, including the use of hand and engine-driven instrumentation techniques are adopted in PNU in similarity with other international dental schools (Al Raisi, Dummer & Vianna, 2019). The emphasis on the inclusion of NiTi engine-driven instruments within the undergraduate dental curriculum to enhance student’s clinical experience and improve the overall treatment care provided has been reported (Martins et al., 2012). Additionally, clinical occurrences of procedural mishaps are undesirable and could negatively impact the overall treatment success (Ng et al., 2008; Cheung & Liu, 2009).

To the best of knowledge of the authors, this study was first conducted in the Gulf Cooperation Countries which looked into both the radiographic occurrence of multiple endodontic mishaps and their correlation with multiple variables in an undergraduate dental clinic utilizing both hand and PTU rotary instrumentation techniques. Overall, results of this study indicate that the first null hypothesis was rejected as the overall occurrence of endodontic mishaps was significantly associated with hand rather than engine-driven rotary instrumentation. Conversely, the second null hypothesis was partially rejected as the occurrence of procedural mishaps positively correlated with the increase in number of canals per tooth and degree of root curvature, however, the operator level and tooth position showed no correlation.

In-line with our finding, Abu-Tahun et al. (2014) also concluded improved performance of NiTi rotary instrumentation in comparison to SS hand files within the hands of dental students. On the contrary, Haug et al. (2018) reported no differences between hand and engine-driven instrumentation techniques in an undergraduate student clinic, however in this study a reciprocation system rather than rotary system was adopted.

A ledge is defined as an artificial created irregularity within the root canal which could impede instrument placement to the full canal length, while a zip is a tear-drop shape apical mishap formed due to instrument extension through the apex which subsequently transports the outer wall (AAE, 2019). In this study, an increase occurrence of ledges was mainly associated with hand file instrumentation and of higher prevalence in curved canals of posterior teeth. These results are in similarity with previous studies (Eleftheriadis & Lambrianidis, 2005; Abu-Tahun et al., 2014). The use of NiTi instruments are reported to reduce canal straightening and provide well centered preparations of curved root canals (Hülsmann, Peters & Dummer, 2005). Likewise, the use NiTi rotary instrumentation improved overall endodontic technical quality and maintained original canal curvature with less canal straightening within the hands of the experienced (Schäfer, Schulz-Bongert & Tulus, 2004), and inexperienced operators (Abu-Tahun et al., 2014).

A perforation is an undesirable, mechanical or pathologic, communication between the internal root canal and the external tooth surface (AAE, 2019). Although this study resulted in a non-significant difference between the instrumentation technique and perforation occurrence, engine-driven rotary instrumentation reduced the perforation occurrence by 35% compared to hand files. Specifically, apical root perforation was the most prevalent type of perforation observed in this study. Over-instrumentation, mainly associated with multi-rooted teeth, was largely seen in these unfortunate scenarios. The high occurrence of root perforation in multi-rooted teeth is in similarity with previous studies (Eleftheriadis & Lambrianidis, 2005; Hendi, Karkehabadi & Eskandarloo, 2018). Although, low occurrence of furcation and strip perforation was recorded in this study. The combined usage of SS Gates-Glidden drills with hand instrumentation could be linked with the increased occurrence of perforation in the hand instrumentation group. Indeed, the occurrence of perforation mishaps has shown a positive correlation with the use of Gates-Glidden drills (Wu, Van der Sluis & Wesselink, 2005). The results of this study also revealed limited occurrence of instrument separation. Within an undergraduate clinical setting instrument separation of SS hand files varies considerably within the literature from 0.5% (Balto et al., 2010) up-to 16.2% of cases (AlRahabi, 2017). More recently, the fracture of two ProTaper file systems (7,993 in total) revealed a very low fracture rate of only 0.37% files (Bruno et al., 2016).

In an attempt to reduce the likelihood of endodontic procedural mishaps, the American Association of Endodontists has released a case complexity evaluation form to assist general dentists and dental students in managing suitable cases within their scope of practice (AAE, 2005). In this study, all clinical cases treated by undergraduate students are initially subjected to case assessment based on American Association of Endodontics case difficulty. Fourth year students treat only minimal difficulty level, while fifth year students treat minimal and moderate difficulty level cases. This initial screening of case difficulty could provide a logical explanation of the overall relatively low occurrence of endodontic mishaps reported in this study.

Furthermore, this study revealed a positive correlation between instrument separation and curved multi-rooted teeth. Indeed, previous literature concludes that the degree of case difficulty is a significant factor that adversely affects the occurrences of endodontic mishaps and the quality of root canal filling within an undergraduate dental clinic (Alsulaimani et al., 2015; Haug et al., 2018). Clinically, the severity of canal infection and the time of treatment at which file separation occurred are important factors that directly affect the treatment outcome (Simon et al., 2008).

The present study revealed no correlation between the undergraduate student level (4^th^ or 5^th^ year) and the occurrence of procedural mishaps. These results are in similarity with published literature (AlRahabi, 2017), and conflicting with others (Balto et al., 2010; Alsulaimani et al., 2015). Furthermore, more cases (*n* = 366) were performed with hand instrumentation in comparison to rotary instrumentation (*n* = 109), as hand instrumentation is the standard instrumentation technique, and the students must show competency in hand instrumentation prior to the use of rotary instrumentation. In summary, within the hands of undergraduate students, preparation of root canals using NiTi engine-driven rotary instrumentation reduced the clinical endodontic mishaps in comparison to SS hand file instrumentation. Furthermore, the degree of case difficulty, specifically the canal curvature was a significant clinical factor linked to mishaps occurrence, particularly ledge formation and instrument separation.

This study has inherent strengths, such as the adoption of two calibrated endodontics for data extraction through electronic patient records and digital radiographic assessment. Data was drawn from an undergraduate clinical setting of a sufficient sample size which aimed to provide a realistic picture of the current clinical status of the primary non-surgical endodontic treated cases. Limitations of the present study such as a retrospective design, data drawn from a single dental school, no clinical information (*e.g.*, tooth survival) and patient-centered information (*e.g.*, occurrence of post-operative pain) are acknowledged by the authors. Moreover, some procedural mishaps cannot be detected on two-dimensional radiographic images, hence remain unaccounted and undetected.

Therefore, further prospective studies and/or randomized controlled clinical trials will be of clinical interest to provide a paucity of evidence and sound conclusions to assist within the undergraduate curriculum development. Additionally, with the constant advancement within the endodontic armamentarium, specifically the NiTi technology, the academic curriculum and clinical training should be regularly monitored and adjusted/upgraded to maximize patient safety and the clinical success. The utilization of case selection assessment forms, such as American Association of Endodontics case difficulty assessment form, seem advantageous in providing suitable cases for the treating undergraduate students, hence reducing endodontic procedural errors.

## Conclusions

The use of NiTi engine-driven rotary instrumentation reduced the clinical endodontic mishaps performed by undergraduate dental students. The degree of canal curvature was a significant clinical factor linked to mishaps occurrence, especially ledge formation and instrument separation.

## Supplemental Information

10.7717/peerj.13858/supp-1 Table S1 Incidence of different perforation type (n[%]) for each tooth

10.7717/peerj.13858/supp-2 Table S2 Incidence of mishaps (n[%]) for each tooth

10.7717/peerj.13858/supp-3 Data S1 Raw data

## Additional Information and Declarations

### Data Availability

The following information was supplied regarding data availability: The raw measurements are available in the Supplemental Files.
