# Supplementary material for: Radiographic assessment of endodontic mishaps in an undergraduate student clinic: a 2-year retrospective study
Source: PeerJ. 2022 Aug 4;10:e13858. doi: 10.7717/peerj.13858 (PMC9357366; doi:10.7717/peerj.13858)
Supplement: Table S1 [file peerj-10-13858-s001.docx]

**Title: Radiographic assessment of endodontic mishaps in an undergraduate student clinic:**

**a 2-year retrospective study**

**Supplementary Table 1: Incidence of different perforation type (n[%]) for each tooth.**

|  | Root perforation | | Strip perforation | | Furcation perforation | |
| --- | --- | --- | --- | --- | --- | --- |
|  | Hand | Rotary | Hand | Rotary | Hand | Rotary |
| 11 | 8[33.3] | 1[50] | 0[0] | 0[0] | 0[0] | 0[0] |
| 12 | 3[30] | 0[0] | 0[0] | 0[0] | 0[0] | 0[0] |
| 13 | 3[18.8] | 0[0] | 0[0] | 0[0] | 0[0] | 0[0] |
| 14 | 13[48.1] | 0[0] | 0[0] | 0[0] | 0[0] | 0[0] |
| 15 | 13[41.9] | 2[66.7] | 0[0] | 0[0] | 0[0] | 0[0] |
| 16 | 4[36.4] | 4[30.8] | 0[0] | 1[7.7] | 0[0] | 0[0] |
| 17 | 1[33.3] | 0[0] | 0[0] | 0[0] | 0[0] | 0[0] |
| 21 | 7[43.8] | 0[0] | 0[0] | 0[0] | 0[0] | 0[0] |
| 22 | 5[31.3] | 0[0] | 0[0] | 0[0] | 0[0] | 0[0] |
| 23 | 3[33.3] | 1[50] | 0[0] | 0[0] | 0[0] | 0[0] |
| 24 | 6[24] | 1[25] | 0[0] | 0[0] | 0[0] | 0[0] |
| 25 | 7[35] | 0[0] | 0[0] | 0[0] | 0[0] | 0[0] |
| 26 | 5[55.6] | 3[37.5] | 0[0] | 0[0] | 0[0] | 0[0] |
| 27 | 1[50] | 0[0] | 0[0] | 0[0] | 0[0] | 0[0] |
| 31 | 3[75] | 0[0] | 0[0] | 0[0] | 0[0] | 0[0] |
| 32 | 2[66.7] | 1[100] | 0[0] | 0[0] | 0[0] | 0[0] |
| 33 | 1[14.3] | 0[0] | 1[14.3] | 0[0] | 0[0] | 0[0] |
| 34 | 4[25] | 2[40] | 0[0] | 0[0] | 0[0] | 0[0] |
| 35 | 6[40] | 1[20] | 0[0] | 0[0] | 0[0] | 0[0] |
| 36 | 7[53.8] | 3[42.9] | 0[0] | 0[0] | 0[0] | 0[0] |
| 37 | 3[42.9] | 2[33.3] | 0[0] | 0[0] | 0[0] | 0[0] |
| 41 | 1[100] | 0[0] | 0[0] | 0[0] | 0[0] | 0[0] |
| 42 | 2[40] | 0[0] | 0[0] | 0[0] | 0[0] | 0[0] |
| 43 | 2[12.5] | 0[0] | 0[0] | 0[0] | 0[0] | 0[0] |
| 44 | 2[20] | 1[16.7] | 0[0] | 0[0] | 0[0] | 0[0] |
| 45 | 7[31.8] | 2[22.2] | 0[0] | 0[0] | 0[0] | 0[0] |
| 46 | 12[57.1] | 7[53.8] | 2[9.5] | 0[0] | 0[0] | 0[0] |
| 47 | 2[33.3] | 1[16.7] | 0[0] | 0[0] | 1[6.25] | 1[16.7] |
| 48 | 1.0[100.0] | 0[0] | 0[0] | 0[0] | 0[0] | 0[0] |
| Total | 134[36.6] | 32[29.4] | 3[0.8] | 1[0.9] | 1[0.3] | 1[0.9] |
