# Supplementary material for: Radiographic assessment of endodontic mishaps in an undergraduate student clinic: a 2-year retrospective study
Source: PeerJ. 2022 Aug 4;10:e13858. doi: 10.7717/peerj.13858 (PMC9357366; doi:10.7717/peerj.13858)
Supplement: Table S2 [file peerj-10-13858-s002.docx]

**Title: Radiographic assessment of endodontic mishaps in an undergraduate student clinic:**

**a 2-year retrospective study**

**Supplementary Table 2: Incidence of mishaps (n[%]) for each tooth.**

| Tooth number | Ledge formation | | Perforations | | Zipping | | Obturation related mishaps | |
| --- | --- | --- | --- | --- | --- | --- | --- | --- |
|  | Hand | Rotary | Hand | Rotary | Hand | Rotary | Hand | Rotary |
| 11 | 0[0] | 0[0] | 8[33.3] | 1[50] | 1[4.2] | 0[0] | 14[58.3] | 1[50] |
| 12 | 0[0] | 0[0] | 3[30] | 0[0] | 0[0] | 0[0] | 3[30] | 0[0] |
| 13 | 0[0] | 0[0] | 3[18.8] | 0[0] | 0[0] | 0[0] | 5[31.3] | 1[33.3] |
| 14 | 2[7.4] | 0[0] | 13[48.1] | 0[0] | 1[3.7] | 0[0] | 15[55.6] | 2[33.3] |
| 15 | 0[0] | 0[0] | 13[41.9] | 2[66.7] | 1[3.2] | 0[0] | 18[58.1] | 2[66.7] |
| 16 | 1[9.1] | 0[0] | 4[36.4] | 5[38.5] | 2[18.2] | 1[7.7] | 6[54.5] | 4[30.8] |
| 17 | 1[33.3] | 0[0] | 1[33.3] | 0[0] | 0[0] | 0[0] | 0[0] | 1[33.3] |
| 21 | 0[0] | 0[0] | 7[43.8] | 0[0] | 1[6.3] | 0[0] | 5[31.3] | 0[0] |
| 22 | 1[6.3] | 0[0] | 5[31.3] | 0[0] | 1[6.3] | 0[0] | 7[43.8] | 0[0] |
| 23 | 0[0] | 0[0] | 3[33.3] | 1[50] | 1[11.1] | 0[0] | 4[44.4] | 0[0] |
| 24 | 1[4] | 1[25] | 6[24] | 1[25] | 1[4] | 0[0] | 15[60] | 0[0] |
| 25 | 1[5] | 0[0] | 7[35] | 0[0] | 0[0] | 0[0] | 7[35] | 1[25] |
| 26 | 0[0] | 0[0] | 5[55.6] | 3[37.5] | 0[0] | 0[0] | 5[55.6] | 6[75] |
| 27 | 0[0] | 0[0] | 1[50] | 0[0] | 0[0] | 0[0] | 1[50] | 1[50] |
| 31 | 0[0] | 0[0] | 3[75] | 0[0] | 0[0] | 0[0] | 0[0] | 0[0] |
| 32 | 0[0] | 0[0] | 2[66.7] | 1[100] | 0[0] | 0[0] | 3[100] | 1[100] |
| 33 | 0[0] | 0[0] | 2[28.6] | 0[0] | 0[0] | 0[0] | 5[71.4] | 0[0] |
| 34 | 0[0] | 0[0] | 4[25] | 2[40] | 1[6.3] | 0[0] | 10[62.5] | 2[40] |
| 35 | 0[0] | 0[0] | 6[40] | 1[20] | 0[0] | 0[0] | 7[46.7] | 1[20] |
| 36 | 3[23.1] | 0[0] | 7[53.8] | 3[42.9] | 0[0] | 0[0] | 7[53.8] | 2[28.6] |
| 37 | 0[0] | 0[0] | 3[42.9] | 2[33.3] | 0[0] | 0[0] | 1[14.3] | 2[33.3] |
| 41 | 0[0] | 0[0] | 1[100] | 0[0] | 0[0] | 0[0] | 1[100] | 0[0] |
| 42 | 0[0] | 0[0] | 2[40] | 0[0] | 0[0] | 0[0] | 2[40] | 0[0] |
| 43 | 3[18.8] | 0[0] | 2[12.5] | 0[0] | 0[0] | 0[0] | 6[37.5] | 0[0] |
| 44 | 0[0] | 1[16.7] | 2[20] | 1[16.7] | 1[10] | 0[0] | 3[30] | 3[50] |
| 45 | 3[13.6] | 0[0] | 7[31.8] | 2[22.2] | 2[9.1] | 0[0] | 12[54.5] | 3[33.3] |
| 46 | 0[0] | 1[7.7] | 12[57.1] | 7[53.8] | 0[0] | 3[23.1] | 10[47.6] | 5[38.5] |
| 47 | 1[16.7] | 0[0] | 2[33.3] | 2[33.3] | 0[0] | 0[0] | 4[66.7] | 3[50] |
| 48 | 0[0] | 0[0] | 1[100] | 0[0] | 0[0] | 0[0] | 1[100] | 0[0] |
